# Retinal Glutamate Neurotransmission: From Physiology to Pathophysiological Mechanisms of Retinal Ganglion Cell Degeneration

**DOI:** 10.3390/life12050638

**Published:** 2022-04-25

**Authors:** Isabella Boccuni, Richard Fairless

**Affiliations:** 1 Institute for Physiology and Pathophysiology, Heidelberg University, 69120 Heidelberg, Germany; 2 Department of Neurology, University Clinic Heidelberg, 69120 Heidelberg, Germany; r.fairless@dkfz-heidelberg.de; 3 Clinical Cooperation Unit (CCU) Neurooncology, German Cancer Consortium (DKTK), German Cancer Research Center (DKFZ), 69120 Heidelberg, Germany

**Keywords:** retina, retinal ganglion cell, glutamate, excitotoxicity, neuronal vulnerability, NMDA receptor

## Abstract

Glutamate neurotransmission and metabolism are finely modulated by the retinal network, where the efficient processing of visual information is shaped by the differential distribution and composition of glutamate receptors and transporters. However, disturbances in glutamate homeostasis can result in glutamate excitotoxicity, a major initiating factor of common neurodegenerative diseases. Within the retina, glutamate excitotoxicity can impair visual transmission by initiating degeneration of neuronal populations, including retinal ganglion cells (RGCs). The vulnerability of RGCs is observed not just as a result of retinal diseases but has also been ascribed to other common neurodegenerative and peripheral diseases. In this review, we describe the vulnerability of RGCs to glutamate excitotoxicity and the contribution of different glutamate receptors and transporters to this. In particular, we focus on the *N*-methyl-d-aspartate (NMDA) receptor as the major effector of glutamate-induced mechanisms of neurodegeneration, including impairment of calcium homeostasis, changes in gene expression and signalling, and mitochondrial dysfunction, as well as the role of endoplasmic reticular stress. Due to recent developments in the search for modulators of NMDA receptor signalling, novel neuroprotective strategies may be on the horizon.

## 1. Retinal Network Integration Requires Glutamate Neurotransmission

Glutamate is the dominant neurotransmitter of the retinal network, where glutamatergic synapses connect the fundamental functional ‘columnar unit’ of the retina, involving photoreceptors (PCs), bipolar cells (BCs) and retinal ganglion cells (RGCs) [1]. Through the ensheathment of this column by Müller glia, glutamate homeostasis and metabolism are finely modulated to enable temporal and spatial control of transmission [2]. This is achieved through the release by Müller glia of glutamate receptor co-agonists such as D-serine [3] and glutamate itself [4], their expression of glutamate and gamma-aminobutyric acid (GABA) transporters [5,6,7], and other roles in the re-uptake, recycling and de novo synthesis of glutamate precursors [8]. Further modulation is achieved through the function of inhibitory interneurons of the retina, horizontal cells and amacrine cells, which help shape glutamatergic neurotransmission through lateral interactions with retinal neurons [9,10,11]. The retinal network is essential to vision since any impairment of this intricate, functional unit inevitably leads to the inability to process visual input.

**Figure 1:**
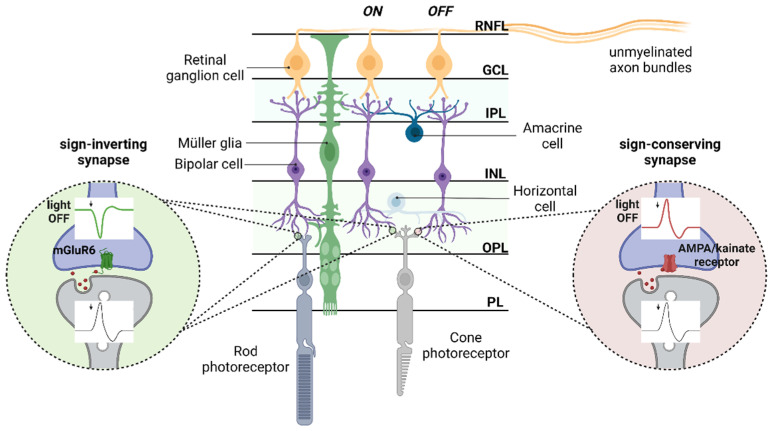
Schematic representation of the retinal network and its different pathways for the processing of visual information. Visual information is processed by the ‘columnar unit’, which includes photoreceptors, bipolar cells, and retinal ganglion cells. Müller glia ensheath the entire ‘columnar unit’ of neurons providing fundamental metabolic and functional support. Horizontal cells and amacrine cells modulate the synaptic signalling between photoreceptors and bipolar cells and between bipolar cells and retinal ganglion cells, respectively. The retinal neuronal layers can be distinguished based on the location of the main cell bodies (photoreceptor layer, PL; inner nuclear layer, INL; ganglion cell layer, GCL) and of the synaptic connections between neurons (outer plexiform layer, OPL; inner plexiform layer, IPL). Retinal ganglion cell axons initially form bundles of unmyelinated fibres at the retinal nerve fibre layer (RNFL), which will then exit the eye and form the myelinated optic nerve. Rod and cone photoreceptors mediate two distinct pathways of light responses: scotopic and photopic vision. In particular, the cone pathway can be additionally distinguished according to ON and OFF, which are interconnected and differentially modulated by glutamate neurotransmission. While the mGluR6 (a metabotropic glutamate receptor) mediates the sign-inverting synapse resulting in rod and ON cone bipolar cell hyperpolarisation in response to glutamate release, the α-amino-3-hydroxy-5-methyl-4-isoxazole propionic acid (AMPA)/kainate receptor mediates the sign-conserving synapse in OFF cone bipolar cells, which depolarise in response to glutamate release under dark conditions.

The ability for the complex information involved in vision to be transmitted using primarily a single neurotransmitter is made possible by several factors. Firstly, multiple types of glutamate receptors are involved, including both ionotropic and metabotropic glutamate receptors (iGluRs and mGluRs) which, in the case of iGluRs, can exist as further variants arising from their existence as co-assemblies of different subunits. The involvement of diverse glutamate receptors can thus determine the differential processing of various components of visual information and has been shown to form the basis of different retinal pathways. As shown in Figure 1, these include pathways responsible for scotopic and photopic vision processed by the rod and cone pathways [12] as well as the processing of light increments and decrements by the ON and OFF pathways [13]. The difference in ON and OFF pathway signals arises primarily from the different glutamate receptors present on ON and OFF BCs. Both rod and cone PCs are depolarized and release glutamate under dark conditions and undergo hyperpolarisation after illumination followed by the reduced release of glutamate [14]. At the next layer, BCs can react in two different ways to changes in photoreceptor activity. OFF cone BCs express ionotropic α-amino-3-hydroxy-5-methyl-4-isoxazole propionic acid (AMPA)/kainate receptors, which permit the entry of depolarising sodium in response to glutamate binding. They are therefore depolarized in the dark (high glutamate release from photoreceptors) and hyperpolarized following light stimuli (reduced glutamate release from photoreceptors), giving rise to a strong signal under dark conditions, contrary to intuition for a sensory system [15,16]. In contrast, ON cone and rod BCs express the metabotropic glutamate receptor mGluR6, which responds to glutamate binding by activating a G-protein coupled signalling cascade leading to the inhibition of inward currents. As a result, the cell is hyperpolarized under dark conditions (high glutamate release) and depolarized under light conditions (low glutamate release). This is termed a sign-inverting synapse since depolarisation of the PCs under dark conditions results in the hyperpolarisation of these BCs and vice versa [16,17].

Secondly, glutamate neurotransmission is finely shaped by the influence of inhibitory and modulatory neurotransmitters such as GABA, glycine, acetylcholine, dopamine, serine, substance P and other neuropeptides [18]. Hence, the glutamatergic retinal network efficiently communicates with the assistance of other neurotransmitters to ensure the best tuning of the visual information.

Furthermore, the existence of ribbon synapses in the retina supports a sustained high basal rate of glutamate release [19]. These synapses are defined by the presence of large proteinaceous ‘ribbons’ at the presynapse, which tether numerous synaptic vesicles to the vicinity of the active zone. This allows their release to be regulated, allowing for both slow, continuous, and fast stimulus-synchronous neurotransmitter release [20]. In addition, a ribbon presynapse can communicate to multiple postsynapses (termed ‘dyads’ and ‘triads’), which due to differential expression of glutamate receptors at the postsynaptic densities, allow the glutamate response to be further tuned [21].

## 2. Distribution of Glutamate Receptors and Transporters in the Retinas

In previous years, several studies implemented different immunohistochemical and electrophysiological techniques in order to map the distribution of glutamate receptors and transporters in the mammalian retina [22,23,24,25]. Table 1 summarises the complex pattern of receptor subunits and transporter subtypes in different retinal cells, which mirrors the intricate mechanisms involved in retinal glutamatergic neurotransmission. Additional and more recent investigations shed light on the genomic and transcriptomic expression of different receptor subunits which appear to be similar in rodent and primate retinas [26,27]. Hence, the following paragraphs aim to depict a general and up-to-date picture of glutamate receptor and transporter distribution according to studies conducted on either rodent or primate retinas.

### 2.1. Ionotropic Receptors

*N*-methyl-d-aspartate (NMDA) receptors are calcium-permeable ion channels which form tetrameric complexes through the combination of two GluN1 with two additional GluN2 or, in some cases, a combination of GluN2 and GluN3 subunits. Due to the diverse combinations of GluN2 (A-D) or GluN3 (A-B) subunits possible, the array of variant NMDA receptors with different functional properties is large [51]. Heterogeneity is further increased by the expression of different splice variants of GluN1. Physiological properties of the ionotropic channel vary in the affinity of their glutamate and glycine-binding sites, deactivation decay time, strength of magnesium blockade, and the desensitization elicited by glycine and calcium. Conventional NMDA receptors contain GluN2 subunits, which have different carboxy–terminal domains that determine the anchoring and trafficking properties of the receptors, such as their membrane localization and downstream signalling [52,53]. Non-conventional NMDA receptors containing a GluN3 (A-B) subunit are subsequently less permeable to calcium, less sensitive to magnesium block, and have emerging roles in developmental synaptic pruning and plasticity [54]. Taken together, the permeability to calcium and the relatively slow decay kinetics of NMDA receptors are key contributors to learning and memory consolidation in the central nervous system (CNS) [55] and temporal resolution in the sensory neurons of the retina [56].

AMPA receptors form heterotetrameric complexes of GluA1-4 subunits [57]. Similar to NMDA receptors, the combination of different subunits determines their heterogeneous physiological properties. Moreover, AMPA receptors are able to interact with many auxiliary subunits which affect their functional diversity, trafficking, and pre- and post-synaptic anchoring [57]. More prone to desensitisation and depression than NMDA receptors [58], AMPA receptors are mainly involved in fast transmission and synaptic plasticity [59] as well as in the spatio-temporal modulation of retinal glutamatergic waves [56,60]. Notably, the GluA2 subunit is typically expressed in neurons and undergoes post-transcriptional editing resulting in a substitution of an arginine (R) residue in place of a glutamine (Q) in the transmembrane portion of the channel. This results in the calcium impermeability characteristic of the GluA2-containing AMPA receptors [61]. However, some atypical AMPA receptors either lack GluA2 or can potentially contain non-edited GluA2 and are subsequently permeable to calcium. These receptors are reportedly expressed only in a few neurons, or under transient conditions, and are interesting for their involvement in development as well as in physiological neuronal plasticity [62] as has been described for retinal neurons [63,64,65]. For a review of physiological functions of calcium-permeable (CP)-AMPA receptors in horizontal, amacrine and retinal ganglion cells, see Diamond, 2011 [65]. Due to the degenerative pathways activated by calcium, CP-AMPA receptors are also particularly relevant under pathophysiological conditions as will be discussed later.

Further ionotropic receptors found in the retina include both kainate receptors and GluD glutamate receptors. Kainate receptors form tetrameric complexes of five subunits (GluK1-5) and, even though their function is not completely understood, they are thought to mediate both the pre- and postsynaptic neuronal responses to glutamate [66]. GluD receptors are formed from GluD1 or GluD2 subunits and were originally characterised as ‘orphan’ receptors with previously unknown endogenous ligands and functions. However, it is now understood that their mutations are strongly associated with cognitive disorders [67]. Although they do not bind glutamate itself, instead being activated by D-serine and cerebellin 1 precursor protein (Cbln1) family members, they exert influence on other glutamate receptors, such as the induction of AMPA receptor internalisation [67,68].

iGluRs are differentially expressed throughout the retina [22,26,29]. PCs express GluN1, GluN2B and GluK5 subunits at their presynaptic terminals [29,30]. Their precise role is not fully understood, although GluK5 has been described to exert a neuromodulatory function as an autoreceptor in rod PCs [28].

Although little is known about glutamatergic responses of horizontal cells, they are thought to be mediated preferentially through AMPA receptors [34,69] (in particular, GluA2-4 subunits), but they do not express any functional kainate receptors [26]. In addition, GluN1 has been shown to be expressed in horizontal cells but, to date, no functional evidence of this NMDA receptor subunit has been reported [29,34]. 

BCs express both AMPA (GluA2-4) and kainate (GluK1-3) receptors [22,37] in different proportions and combinations depending on the functional pathway in which the cell subtype is involved. Notably, a distinct expression of kainate receptors (GluK1) has been described for the OFF-BCs [36]. While BCs express NMDA receptors (GluN1,2C-D) [23,37], their activity has been associated with the rod but not the cone pathway [70]. In particular, presynaptic terminals of rod BCs selectively express the NMDA receptor subunit GluN2D with a potential neuromodulating role of NMDA receptors within the inner plexiform layer (IPL) [38]. 

Amacrine cells express more or less the wide repertoire of iGluRs to different degrees across their over sixty cell subtypes [71]. For example, amacrine cell subtypes AII and AI at the dyad synapse with rod BCs have been shown to express different AMPA and kainate receptors—in particular, the former express GluA2-4 AMPA receptor subunits, whilst the latter express GluD1-2 subunits in combination with GluK2-3 kainate receptor subunits [42]. Amacrine cells also express NMDA receptor subunits (GluN1,2A-C) [29,37], though the differential distribution of GluN2A and GluN2B subunits within the IPL indicate their involvement in different retinal circuits [29]. For instance, glutamatergic amacrine cells show predominant expression of the GluN2A subunit, whereas cholinergic amacrine cells express GluN2B [26]. Extrasynaptic GluN2B-containing NMDA receptors are found also to modulate the glycinergic AII amacrine cell response in the cone pathway, whereas GABAergic A17 amacrine cells express GluN2A-containing NMDA receptors which mediate rod pathway neurotransmission [72]. 

RGCs show predominant expression of NMDA (GluN1,2A-C), AMPA (GluA1-4) and kainate receptors (GluK2-5) [22,26,29,37] with differential synaptic and extrasynaptic localization. In particular, non-NMDA receptors are believed to be the main responders to the synaptic quantal release of glutamate [73,74,75], whereas NMDA receptors are mainly located at peri- and extrasynaptic sites, where they react to ambient glutamate levels and, hence, their activity is enhanced by reductions in glutamate uptake [76,77]. In any case, NMDA receptor activity is shown to contribute to the baseline noise and conducting properties of RGCs under resting conditions [78]. The NMDA receptor subunits are also associated with different scaffolding proteins. For example, GluN2A and GluN2B have been shown to interact with postsynaptic density-95 (PSD-95) at the postsynapses of OFF-RGCs and with synapse-associated protein 102 (SAP102) at perisynaptic sites of ON-RGCs, respectively [79]. In addition, RGCs express the GluN3A subunit in rodents which has been shown to reduce NMDA receptor-induced calcium rises in neurons and to shape small components of the light response [45].

Interestingly, in addition to neurons, ionotropic glutamate receptors are also expressed by retinal glia, with Müller glia expressing AMPA receptors [46] and NMDA receptors [46,47]. However, this expression has mainly been inferred from functional studies of in vitro cultured Müller glia, with immunocytochemical evidence (except for the expression of GluA4 [22]) still lacking. Nevertheless, iGluR expression appears to confer glutamate sensitivity to Müller glia, allowing them to respond to neuronal activity, and to actively participate in retinal glutamatergic transmission. Indeed, not only in vitro stimulation of iGluRs on Müller glia promotes glutamate reuptake [46], but also modulates neuronal excitability by coupling intracellular calcium waves between Müller glia and retinal neurons in both rat [4] and zebrafish [80] studies.

### 2.2. Metabotropic Receptors

mGluRs are members of the family of G protein-coupled heptahelical membrane proteins, associated with either the inhibition or activation of different downstream signalling pathways [81]. According to sequence homology and function, they are classified into three subclasses: Group I (mGluR1,5), Group II (mGluR2,3) and Group III (mGluR4,6,7,8) [81]. In general, their activation is thought to modulate the glutamate feedforward neurotransmission of the retinal network [27]. Retinal neurons can each express multiple members of the metabotropic receptor family, with the exception of mGluR3 [23,27].

mGluRs are expressed as autoreceptors in the presynaptic terminals of PCs, BCs, horizontal and amacrine cells and at postsynaptic terminals of BCs, RGCs, horizontal and amacrine cells. mGluRs are thought to play a fundamental role as glutamate sensors at the ribbon synapse, where their activation leads to a decrease in presynaptic terminal calcium levels, and subsequently a reduction in glutamate release [23,31]. Group III receptors (in particular, mGluR8) are expressed on PC and BC presynaptic terminals and amacrine cells and are likely to shape different aspects of the light response, including the light processing differentiation of the ON and OFF pathways [31,39,40,82]. As mentioned earlier, mGluR6 mediates the sign-inverting response at postsynaptic terminals of ON-BCs coupling glutamate release in dark conditions with the hyperpolarisation of ON-BCs [17,27]. At the ‘dyad’ ribbon synapse between a BC presynaptic terminal and RGC and amacrine cell postsynaptic terminals, the differential expression of glutamate receptors asymmetrically modulates the glutamate release and response. Indeed, different amacrine cells express a different repertoire of mGluRs, which finely tunes the glutamate response giving rise to a specific translational component of the visual information [83]. For instance, mGluR2 expressed by starburst amacrine cells shapes the directional sensitivity of the light response by modulating GABA and glycine release at their synapses with RGCs [43]. Group I and Group III receptors are also expressed by amacrine cells, but very little is known about their specific signalling cascades. 

Although RGC function is dominated by iGluRs, they also express all the mGluRs (except for mGluR3) at their postsynaptic terminals in different numbers and combinations depending on their developmental stage and the RGC subtype [23,84]. Of note, there is not much overlap in the expression of different mGluRs at the IPL, where the dendrites of the ganglion cells are located, suggesting their discrete roles in shaping different processing of the visual input [23,40,82]. For instance, the sustained versus the transient response to light by RGCs originates at the BC synapse and is partially defined by the different kinetics of the mGluRs expressed at BC presynaptic sites located in the different sublaminae of the IPL [85]. 

### 2.3. Glutamate Transporters

In addition to glutamate receptors, glutamate transporters also play an important role in shaping glutamate-mediated transmission by modulating temporal and spatial aspects of glutamate signalling and by regulating its clearance from the extracellular space. The sodium-coupled excitatory amino acid transporters (EAATs) can be distinguished into five subtypes (EAAT1-5) relevant for both neuronal and glial glutamate reuptake [86], which display different glutamate transportation rates and are differentially expressed across the retina. 

EAAT1 (or GLAST) is solely expressed by Müller glia, displaying a strong immunoreactivity within the whole retinal tissue where the glial cells ensheath all retinal neurons [24,32,48]. Interestingly, EAAT1 has also been shown to be expressed on the endothelial cell membrane of retinal capillaries which contributes to glutamate reuptake in the inner retina [87]. EAAT2 (or GLT-1) is expressed by PCs and by different subtypes of BCs and amacrine cells [24,32]. EAAT3 (or EAAC1) is found on RGCs, horizontal cells as well as various BCs and amacrine cells [32,35]. The expression of EAAT4 colocalizes with EAAT1 in retinal astrocytes [49]. Interestingly, EAAT5, which is only expressed at very low levels in the CNS, is much more predominant in the retina (in rod PCs and BCs [33]). Genetic ablation of EAAT1 in the retina, as well as pharmacological inhibition of EAAT1 and EAAT2, leads to both a significant increase in extracellular glutamate and RGC death, with only insufficient compensatory activity of the other transporters [88,89], indicating that the glutamate reuptake dynamics and the specific localization of these transporters are critical for spatio-temporally efficient glutamate clearance. Indeed, while EAAT1-3 have relatively fast reuptake kinetics, ensuring a rapid glutamate turnover rate [90], both EAAT4 and EAAT5 display reduced glutamate transporter activity compared to the other family members. In addition to the coupled sodium conductance, EAATs display uncoupled chloride conductance [91], and in particular, EAAT4 and EAAT5 have a high chloride conductance which suggests their potential roles as inhibitory modulators [92,93,94]. Indeed, recent findings have shown that EAAT5 functions as an inhibitory autoreceptor in rod PCs [95,96], and electrophysiological analysis of EAAT5 knock-out retina revealed its role in shaping the temporal resolution of the light response [97,98]. In addition to EAATs, Müller glia also express the cysteine-glutamate transporter (xCT) [50] which is particularly involved in the response to oxidative stress.

The combined elements of different glutamate receptors and transporters function in a complex overlay to mediate and modulate glutamate transmission in the retina. However, as outlined below, the visual system is also vulnerable to disease and injury, where a perturbation in glutamate signalling is increasingly understood to be a contributing factor.

## 3. Vulnerability of the Retina and RGCs to Disease

The visual system is vulnerable to many different pathophysiological conditions with evidence of retinal degeneration occurring as a result of glaucoma [99,100], diabetic retinopathy [101,102], retinal ischemia [103], and optic neuritis [104,105]. In addition, retinal degeneration is also reported in other neurodegenerative diseases, traditionally affecting other CNS regions, such as multiple sclerosis (including in the absence of associated optic neuritis) [106,107,108], Alzheimer’s [109,110], and Parkinson’s [111,112], diseases, giving rise to the concept that the retina can serve as ‘diagnostic window into the brain’ and giving insight into neurodegenerative processes occurring elsewhere [113]. In humans, thinning of the retinal nerve fibre layer (RNFL) containing RGC axons has been used as a diagnostic indicator of retinal, and particularly RGC, degeneration (as well as testing of neuroprotective therapies) since this region is particularly vulnerable in glaucoma [114], diabetic retinopathy [115], retinal ischemia [116] and in optic neuritis [104] as well as in multiple sclerosis [117], Alzheimer’s [118,119] and Parkinson’s [120,121] diseases.

This vulnerability may, in part, reflect the unique anatomical structure of the retina. For example, the optic nerve head (ONH), where RGC axons exit the retina to form the optic nerve, is highly exposed to biomechanical stress due to the intrinsic flexibility of this area which allows eye mobility in the orbit [122]. In addition, this area also has an atypical blood–brain barrier [123,124] which may be more permeable to early circulating factors that lead to degeneration. RGC axons, which connect the retina to the higher visual regions of the CNS, pass through the RNFL towards the optic disc, where they make an abrupt 90-degree turn before passing through the lamina cribrosa, from where they make long projections along the optic nerve to the brain. Although the optic nerve is a myelinated tract, most RGC axons remain unmyelinated within the RNFL, which allows light to penetrate through this layer to the photoreceptor layer. This lack of myelin, however, results in a high energy demand for fast transmission of action potentials [125,126], which in turn may render RGCs more susceptible to mitochondrial dysfunction and oxidative stress, as has been described for other diseases [127]. Collectively, these architectural peculiarities contribute to the vulnerable position of RGCs, which have been described as the ‘most vulnerable of all neuronal cell types’ [125]. 

In addition to this anatomical exposure, the intrinsic properties of RGCs may also explain their high susceptibility to degenerative processes. This is reflected by the differential susceptibility of RGC subtypes to various diseases. Given the complexity of the retinal network, around thirty subtypes of RGCs have been classified based on their morphology (such as soma size and dendritic arborisation), the functional pathways in which they are involved, and their molecular signatures [128]. RGC synaptic loss and dendritic shrinkage following injury have been shown to be subtype-specific [129,130]. For instance, in glaucoma, a reduced density of ribbon synapses occurred specifically in OFF-RGCs accompanied by a loss of their dendritic arborisation and spontaneous synaptic activity [131,132,133]. In particular, αRGCs and direction-selective RGCs appear to be selectively vulnerable, whereas intrinsically photosensitive RGCs (ipRGCs; a distinct type of retinal neurons mediating adaptive light responses) display some resilience in the glaucomatous optic nerve crush disease model [134,135]. αRGCs have a larger soma size than other RGC subtypes [136] and may be particularly vulnerable to NMDA-induced degeneration, as suggested by a study reporting this to affect predominantly the larger RGCs [137]. However, in contrast, another study suggested that smaller RGCs located in the retinal centre were more vulnerable to NMDA [138] and other studies identify αRGCs as a more resistant cell type following axotomy [139]. Collectively, these data emphasise the importance of considering both the precise kind of insult to—as well as the retinal location of—RGCs in order to appreciate the vulnerability of the different subtypes. 

Amongst the αRGCs, αOFF-RGCs were reported to be more vulnerable to degeneration in models of both glaucoma [133] and optic neuritis [140], compared to αON-RGCs. Reasons for this may reflect their pacemaker-like electrophysiological characteristics, arising from the higher sodium and calcium membrane conductance of αOFF-RGCs under resting conditions in comparison to the αON-subtype [141,142]. In glaucoma, αOFF-RGCs also express higher numbers of GluA2-lacking CP-AMPA receptors, which may make them more prone to detrimental high calcium influx [143,144]. Moreover, their dendrites extend into the outer OFF sublamina, which is actually more exposed to blood capillaries distributed at the interface between the inner nuclear layer and the IPL [145]. This may underlie their differential vulnerability to systemic excitotoxic insults. Conversely, in hyperglycaemic conditions, it has been shown that αRGCs and melanopsin-positive ipRGCs that stratify to the inner ON sublamina undergo remodelling of their dendritic arbour as a possible result of adaptive neuroprotection [146,147].

Despite the different disease conditions (including both retinal and non-retinal pathologies), which can lead to degeneration of the retina in general, and RGCs in particular, they may be connected by a common mechanism involving glutamate excitotoxicity [148,149] that either initiates or leads to an exacerbation of retinal degeneration.

## 4. Glutamate Excitotoxicity in the Retina

Although glutamate signalling and homeostasis are essential for the transmission and modulation of visual signals across the retina, their dysregulation has also been linked to degenerative processes in several retinal pathologies such as glaucoma [99,100], diabetic retinopathy [101,102], retinal ischemia [103], and optic neuritis [105]. The first reports that glutamate can cause neurodegeneration are in fact derived from studies of the retina following subcutaneous injection in mice [150] and intravitreal injection in rats [151]. It has since been shown that the application of glutamate can mimic the pathophysiology of different retinal diseases. However, increases in extracellular glutamate have not consistently been detected in retinal pathologies, such as in glaucoma, where no reliable evidence of glutamate increases has been seen [152]. Reasons for this may involve spatially restricted glutamate elevations which do not occur at the global level but may also result from alterations in glutamate receptors affecting neuronal sensitivity to glutamate or imbalances in the protective and degenerative signalling pathways elicited.

### 4.1. Impairment in Glutamate Clearance and the Role of Müller Glia

Evidence that deficient glutamate clearance is involved in glutamate-mediated excitotoxicity includes observations that exogenous application of glutamate alone does not elicit the same degree of retinal degeneration as that observed in disease models with an equivalent level of glutamate elevation [153]. Similarly, the application of NMDA, which is impervious to endogenous clearance mechanisms, results in greater degeneration [154,155]. Further evidence of the role of glutamate transporters includes the reduction or loss of function of glutamate transporters in several retinal diseases, such as diabetic retinopathy [156,157], glaucoma [158,159], and retinal ischemia [160]. Conversely, an increased expression of glutamate transporters has been described in other conditions such as optic neuritis [161], where glutamate excitotoxicity is linked to neuroinflammation. This may represent a compensatory mechanism occurring also in other brain areas in multiple sclerosis [162,163], though it may ultimately fail since increased retinal glutamate levels have been reported in multiple sclerosis [164].

A further indicator of the role of glutamate transporters is the ubiquitous involvement of Müller glia, which provide metabolic support to neurons and are primarily responsible for neurotransmitter homeostasis. The health of both neurons and the retinal vasculature depend upon efficient Müller glial function [165]. In turn, Müller glial malfunction is one of the major initiating and contributing factors that lead to impaired glutamate homeostasis and, as a result of ensuing increases in endogenous glutamate, to a collapse of retinal function [166]. Although in some instances, Müller glia can exert a neuroprotective influence during initial disease phases, at later stages, their gliotic reaction can lead to an imbalance in glutamate homeostasis exacerbating neurodegeneration [166,167,168,169].

Mechanisms leading to impaired glutamate clearance can be manifold. One mechanism is a collapse in the sodium gradient required for sodium-dependent glutamate transportation. This gradient is maintained by the activity of the sodium/potassium-ATPase which is, of course, sensitive to ATP depletion upon impairment in energy metabolism. This can occur, for example, due to a lack of oxygen in retinal ischemia, or as a result of mitochondrial damage in glaucoma [170,171]. Mechanisms to protect against this include ‘metabolic symbiosis’ between RGCs and Müller glia, whereby they can exchange lactate as a substitute for glucose as an oxidative mitochondrial metabolic substrate during conditions of hypoglycaemic and oxidative stress [172]. However, prolonged starvation can override this protective mechanism, resulting in the increased susceptibility of Müller glia to oxidative stress, which results in mitochondrial failure and downregulation of glutamate transporters [173]. In particular, oxidative stress induces downregulation of GLAST and an upregulation of xCT, which has a fundamental role in releasing glutathione, an important antioxidant molecule [174,175,176].

In addition to glutamate clearance, Müller glia are also involved in mediating the interface between the retinal vasculature and retinal neurons [177]. They not only facilitate the importation of circulating nourishing factors, but they also secrete different growth factors—such as vascular endothelial growth factor (VEGF)—and cytokines—including interleukin-1β (IL-1β) and tumour necrosis factor-α (TNF-α)—in response to vascular and neuronal changes [178]. These molecules are described to have dual effects by enhancing either protective or detrimental pathways. For instance, VEGF release, particularly the isoform VEGFA [179,180], in combination with reactive oxygen species (ROS) production in diabetic retinopathy has been shown to enhance retinal degeneration [181], whereas VEGFD is protective for RGCs and the retinal vasculature [182]. In addition, the secretion of TNF-α has been shown to exert early neuroprotective effects [183] but to ultimately induce Müller cell gliosis and RGC death in the glaucomatous retina [184]. Upon a neuroinflammatory insult, the quiescent and neuroprotective Müller glia undergo reactive, proliferating gliosis [185], which in turn enhances pathological angiogenesis [186,187] and increases RGC vulnerability to retinal injury [169]. Interestingly, treatment with dexamethasone, which lowers levels of IL-1β and TNF-α, is protective against hyperglycaemia for RGCs but not for the Müller glia [188], suggesting that once gliosis occurs, it is not easily reversed. Therefore, Müller glia have a critical role at the neurovascular interface, and the pathological and potentially irreversible changes occurring during reactive gliosis have been shown to lead to severe impairment of glutamate clearance by inducing changes in glial glutamate metabolism and their expression of glutamate transporters [159,161,189].

### 4.2. Glutamate Receptor-Induced RGC Degeneration

Toxic glutamate effects are mediated by glutamate receptors, which in the retina, as discussed below in this review, predominantly involve NMDA receptors. However, there is some evidence that non-NMDA receptors can also mediate glutamate excitotoxicity. For instance, a general increase in expression of both NMDA (GluN1) and AMPA (GluA2-3) receptor subunits has been shown in hypoxic-ischemic retinas [190]. Furthermore, prolonged activation of AMPA/kainate receptors can lead to amacrine and RGC degeneration in the chick retinal embryo [191]. As mentioned earlier, retinal neurons can express both calcium-permeable and calcium-impermeable AMPA receptors, and this balance may influence their susceptibility to degeneration. For example, it has been shown that modelling of glaucoma through increased pressure leads to an increase in the expression of CP-AMPA receptors in RGCs both in vitro [143] and in vivo [144]. This latter study also suggested a role for Müller glial-derived TNF-α in driving this expression and demonstrated the degenerative contribution of these CP-AMPA receptors through the application of selective blockers. This same mechanism may even underlie NMDA receptor toxicity since the application of NMDA also promoted Müller glial production of TNF-α, resulting in an increase in CP-AMPA receptors [144,192]. Metabotropic receptors might also be involved in mediating glutamate excitotoxicity since the systemic application of a blocker of mGluR1 was able to block this, also supported by the lack of RGC degeneration following glutamate application in mice lacking mGluR1 [193]. 

Nevertheless, the majority of studies have implicated the NMDA receptor as the main mediator of glutamate excitotoxicity in the retina, such as in glaucoma [194,195,196], ischemic [190,197,198], and diabetic retinopathy [101,199,200], as well as optic neuritis [105]. This receptor, however, can also activate survival pathways, which may promote neuroprotective mechanisms; therefore, attempts at resolving this discrepancy depend upon the elucidation of the divergent downstream pathways activated under different conditions [201].

### 4.3. Factors Affecting NMDA Receptor-Mediated Degeneration

It has become increasingly clear that the concept of NMDA receptor over-activation does not adequately explain the initiation of neurodegeneration. Rather, the localisation and subunit composition of these receptors play an important role in determining the neuronal fate and must be considered. NMDA receptors can exist both within the postsynaptic density, as well as in perisynaptic and extrasynaptic locations. Several studies have demonstrated that activation of extrasynaptic NMDA receptors can initiate degenerative cascades, whereas it is the activity of synaptic NMDA receptors that leads to the promotion of neuroprotective mechanisms [201]. However, how distinct signalling cascades are activated is currently unclear, though it may involve differential coupling of incoming calcium with cellular organelles, such as mitochondria and endoplasmic reticulum (ER), which are located near these calcium hotspots. Alternatively, NMDA receptors may interact with different scaffolding or downstream signalling proteins depending upon their location, as has recently been described for transient receptor potential cation channel subfamily M, member 4 (TRPM4) [202]. This location-dependent interaction may reflect different protein compositions associated with synaptic and extrasynaptic regions, or, alternatively, may reflect different NMDA receptor subunit compositions in these regions. Interestingly, GluN2A and GluN2B have been proposed to be associated with synaptic and extrasynaptic locations of NMDA receptors, respectively, and therefore may couple NMDA receptor activation with different downstream factors resulting in different cell fates [203,204]. In RGCs, extrasynaptic NMDA receptor activation may explain some of their vulnerability to degeneration since they have been reported to contain large pools of extrasynaptic NMDA receptors [77], which in hippocampal neurons were associated with degenerative processes [205]. In addition, pharmacological inhibition of the extrasynaptic NMDA receptor subunit GluN2B (as well as GluN2D) has been reported to be neuroprotective against retinal glutamate excitotoxicity [206]. GluN2B has also been implicated in RGC degeneration in glaucoma since its expression is enhanced in this disease [207]. 

Of note, pathological NMDA receptor activation affects Müller glial function and retinal vasculature permeability to immune cell infiltration. Several studies have reported that endogenous glutamate clearance and synthesis, when impaired, lead to severe damage to the inner layers of the retina mainly through NMDA receptor-mediated intracellular calcium increases [208,209,210]. Conversely, NMDA receptor stimulation increases the uptake activity of glutamate transporters on Müller glia [46], indicating that there is bidirectional communication between retinal neurons and Müller glia. Additionally, NMDA receptors have been described to be an important link between glutamate excitotoxicity and neuroinflammation in neurodegenerative diseases [148]. For example, endothelial cells have been reported to express functional NMDA receptors that, if overstimulated by a glutamate excitotoxic insult, enhance immune infiltration by disrupting the blood–brain barrier in multiple sclerosis [211], which might imply a similar alteration during optic neuritis. Furthermore, NMDA receptor activity can influence Müller glial synthesis and secretion of VEGF, thus functioning as a modulator of endothelial cell proliferation and angiogenesis. Under hyperglycaemic and hypoxic conditions in vitro, this control mechanism has been shown to become impaired [47]. In the reverse direction, following immune infiltration, resident microglia become activated and interact with Müller glial cells exacerbating the overall pathophysiological response [212]. In particular, NMDA-induced toxicity in the retina has been shown to enhance inflammatory activity and the secretion of the cytokine IL-1β, which can act as an initiator of the apoptotic cascade [213]. 

A growing body of scientific evidence has shed light on the key pathophysiological role of NMDA receptors in the degeneration of RGCs, whose intricate downstream mechanisms of action are elucidated in the following section. 

## 5. Cellular Mechanisms of NMDA Receptor-Induced Excitotoxicity in RGCs

**Figure 2:**
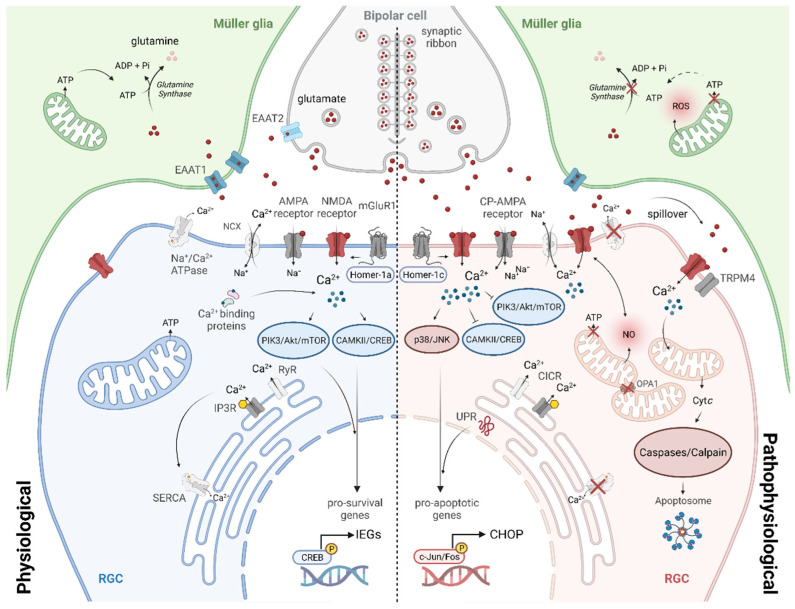
Schematic comparison of the physiological and pathophysiological cellular mechanisms induced by glutamate release at the retinal ganglion cell (RGC) synapse. In response to light stimulation, glutamate is readily released from the presynaptic ribbon of bipolar cells onto their postsynaptic partners. Under *physiological* conditions, the initial AMPA receptor-mediated membrane depolarization increases the opening probability of the NMDA receptors, inducing a temporary intracellular calcium influx. Calcium can be also released through inositol 1,4,5-trisphosphate receptors (IP3R) and the ryanodine receptors (RyR) expressed on the endoplasmic reticulum membrane. Functional mitochondrial metabolism efficiently contributes to intracellular calcium homeostasis, for instance, through the supply of ATP allowing calcium reuptake by Sarco-Endoplasmic Reticulum Calcium ATPase (SERCA) and extrusion of calcium to the extracellular space through the sodium-calcium ATPase. In addition, calcium is extruded by the sodium-calcium exchanger (NCX) using the physiological sodium gradient, and calcium-binding proteins buffer calcium, limiting its spatio-temporal availability. mGluR1 is associated with the scaffolding protein Homer-1a, which can, together with NMDA receptor-mediated synaptic calcium influx, contribute to pro-survival signalling pathways including PIK3/Akt/mTOR and CAMKII/CREB. Phosphorylated CREB translocates to the nucleus where it functions as a transcription factor of the immediate early genes (IEGs). Glutamate reuptake by EAAT1 expressed on Müller glia and by EAAT2 expressed on bipolar cells, allows for rapid glutamate clearance at the synaptic cleft. Physiological glutamate metabolism in Müller glia allows recycling of the neurotransmitter by glutamine synthase which transforms glutamate to glutamine using ATP. Prolonged exposure to glutamate under *pathophysiological* conditions, induces intracellular calcium overload by overstimulating glutamate receptors including calcium-permeable (CP)-AMPA receptors and destabilising the membrane potential resulting in reverse activity of NCX. Prolonged NMDA receptor-mediated calcium influx, and mGluR1 interaction with the isoform Homer-1c, cause calcium-induced calcium release (CICR) from endoplasmic reticulum calcium stores, exacerbating calcium dyshomeostasis and triggering mitochondrial dysfunction and energy failure. Hence, reduced ATP availability limits the capability of SERCA and the sodium-calcium ATPase to extrude intracellular calcium. In addition, NMDA receptor overstimulation leads to mitochondrial fragmentation and to the production of nitric oxide (NO) which in turn both enhance NMDA receptor activity. Calcium overload and the unfolded protein response (UPR) promote the pro-apoptotic signalling pathway of p38/JNK and inhibit pro-survival ones, and the phosphorylation and activation of the c-Jun/Fos transcription factor results in transcription of pro-apoptotic genes such as C/EBP Homologous Protein (CHOP). Under pathophysiological conditions, glutamate clearance is impaired because of downregulation of Müller glial glutamate transporters and their metabolic failure, resulting in ROS production and reduction of mitochondrial ATP production. This results in glutamate spill-over from the synaptic to the extrasynaptic space, activating extrasynaptic NMDA receptors and promoting neuronal degeneration through a myriad of downstream mechanisms [214]. These include calpain activation and activation of the caspase-mediated apoptotic pathway as a result of cytochrome *c* (Cyt*c*) release from mitochondria.

Downstream of glutamate receptor over-activation, neurodegenerative processes are initiated through an initial influx of primarily calcium, leading to subsequent alterations in energy metabolism, protein folding and changes in gene expression which form the main hallmarks of glutamate excitotoxicity. Figure 2 summarises these mechanisms which have been intensively studied over decades. Recent discoveries show how homeostatic mechanisms eventually fail and how these processes are interconnected. In this review, NMDA receptor-dependent cellular mechanisms of excitotoxicity are described in the context of RGC degeneration, whose vulnerability is reflected also in the interdependence of different pathways to maintain and promote neuronal survival. As described before, Müller glial function is also altered upon exposure to high levels of extracellular glutamate and significantly contributes to degeneration subsequent to pathological and prolonged NMDA receptor activation.

### 5.1. Intracellular Calcium Dysregulation

Under physiological conditions, synaptic glutamate release initially activates AMPA/kainate receptors, which induce an initial membrane potential depolarization that increases the opening probability of NMDA receptors through the removal of the voltage-dependent magnesium block [215]. Calcium ions are then driven by their high concentration and potential gradients into the intracellular space. In RGCs, NMDA receptors are the major source of this calcium influx, with partial contributions from the voltage-gated calcium channels [216,217]. In fact, GluN1, GluN2A-D, and GluN3A subunits of NMDA receptors are upregulated within three hours, following a hypoxic insult to RGCs [218]. Other potential sources of toxic calcium in RGCs include kainate receptors [219,220], as well as GluA2-lacking CP-AMPA receptors, as has been shown in the glaucomatous retina [143,144]. Prolonged and pathological exposure to glutamate of RGCs in culture has been shown, similar to other neuronal subtypes, to lead to a dysregulation in calcium homeostasis, also known as ‘dysregulated calcium dynamics’ (DCD) [221]. Here, the inability of RGCs to sufficiently buffer or extrude excessive calcium ions leads to a breakdown in the homeostatic machinery resulting in a secondary, irreversible calcium increase which ultimately leads to cell death. Although the underlying mechanisms have not been clearly elucidated in RGCs, they are similar to reports from hippocampal neurons and may involve reverse activity or calcium-mediated cleavage of the sodium-calcium exchanger (NCX) [222,223]. Support for this includes in vivo evidence of NCX involvement in NMDA receptor-mediated excitotoxicity in retinal ischemia [224]. In addition, NMDA receptor activation leads to an elevation in intracellular calcium in RGCs through a mechanism called ‘calcium-induced calcium release’ (CICR) [225]. CICR has been described in neurons to exacerbate glutamate-induced intracellular calcium dysregulation via saturation of internal calcium buffers in various neurodegenerative diseases [226]. In particular, the calcium stored in the ER is released in response to the activation of inositol 1,4,5-trisphosphate receptors (IP3Rs) and ryanodine receptors (RyRs) [227], a process regulated by mitochondrial activity [228,229]. 

To protect against these processes, intracellular calcium homeostasis is maintained by calcium buffering, which can be achieved by calcium-binding proteins coupled to various subunits of the ionotropic glutamate receptors through an array of scaffolding proteins and cytoskeletal components [230]. This may explain why some neurons have been shown to be more susceptible to calcium deriving from the addition of a chemical ionophore than glutamate receptor-derived calcium since calcium buffering is maintained in microdomains surrounding glutamate receptor entry points [231]. One scaffolding protein implicated in the mediation of neurodegeneration is Homer-1, a component of the retinal glutamatergic postsynaptic density involved in mediating downstream intracellular signalling. Homer-1 can form protein complexes with both NMDA receptors and type 1 mGluRs, as well as various other scaffold proteins such as PSD-95 [232,233]. It has been shown in mouse cerebellar neurons that mGluR1 can inhibit NMDA receptor activity through the activity of the Homer-1a isoform, which promotes cell survival and synaptic plasticity [234]. Indeed, while Homer-1a has been demonstrated to promote RGC survival following ischemic retinopathy [235], Homer-1c was shown to be upregulated in the retina of DBA/2J mice and to correlate with disease severity [236]. Its upregulation served as an early marker of disrupted synaptic connectivity and degeneration and was suggested to potentially link synaptic activity to enhanced internal calcium release via the IP3Rs expressed on the ER [236,237]. This differential coupling of iGluRs to intracellular buffering complexes may also explain why the NMDA receptor subunits GluN2A and GluN2B have been associated with differential susceptibility to glutamate toxicity [238,239]. As mentioned earlier, in RGCs the GluN2A and GluN2B subunits of NMDA receptors have been shown to preferentially associate with PSD-95 and SAP102 scaffold proteins, respectively [79]. These subunits are also associated with NMDA receptors found in different localisations in neurons [240]. This is also true in RGCs with GluN2A being associated with synaptic, and GluN2B with perisynaptic sites [79]. Thus, it may be that calcium entering by synaptic NMDA receptors may be more readily buffered, compared to extrasynaptic NMDA receptor-derived calcium, due to differences in their intracellular binding partners. Conversely, mitochondrial uptake of calcium has been shown to lead to both metabolic impairment and apoptotic signalling (see below), and since mitochondria are found outside of postsynaptic dendritic spines [241], this may be an alternative explanation for the synaptic/extrasynaptic NMDA receptor divergence in downstream signalling. 

Different calcium buffering capacities may underlie the differential susceptibility of various neuronal cell types to glutamate excitotoxicity, including RGC subtypes. The varying expression profile of different members of the large calcium-binding protein family may influence the risk of neurodegeneration due to their varying kinetics and subcellular localization which define neuronal calcium-buffering capacities [242,243]. Indeed, calcium-binding proteins are proposed to contribute to neuronal-subtype susceptibility to glutamate-induced degeneration where parvalbumin expression, as well as being a marker of vulnerable interneurons of the hippocampus, is also associated with a predisposition to neurodegeneration in retinal neurodegenerative diseases [244]. In a rodent model of retinal ischemia, it has been reported that parvalbumin-positive RGCs were more susceptible to cell death than calbindin-expressing cells [245,246]. Moreover, an overall reduction in parvalbumin expression in the retina was also shown in diabetic retinopathy [247] and glaucoma [248], suggesting the possible selective loss of parvalbumin-positive neurons. Similarly, αON-RGCs, which are reportedly more resistant to degeneration in optic neuritis [140] and glaucoma [133], express calbindin, unlike the susceptible αOFF-RGCs [249]. However, the picture is more complex than this due to the presence of multiple calcium-binding protein family members in retinal neurons [250] and because the expression profile might only be indirectly associated with neuronal vulnerability, being more closely coupled with the firing kinetics and other functional characteristics of the neurons. 

Collectively, NMDA receptor-mediated calcium elevations in RGCs can activate a range of downstream death signalling mechanisms, which will be explored in more detail in the following paragraphs.

### 5.2. Imbalance of Signalling Pathways and Gene Expression

In response to synaptic NMDA receptor-induced calcium influx in RGCs, similar to other neurons, the pro-survival calcium/calmodulin-dependent protein kinase II (CAMKII), particularly the isoform CAMKIIαB [251], is activated resulting in the phosphorylation of the cyclic AMP-response element binding protein (p-CREB) [252]. As a result, p-CREB translocates to the nucleus where it acts as an important transcription factor of an array of survival genes, termed the immediate early genes (IEGs). Collectively, this pathway leads to the activation of a protective neuronal response [253], which has been reported in the retina following neurotoxic insults [254]. For instance, p-CREB promotes the upregulation of brain-derived neurotrophic factor (BDNF) and its secretion from Müller glia and retinal neurons with a fundamental neuroprotective function for RGCs [255]. BDNF binds the tropomyosin receptor kinase B (TrkB) receptor on RGCs and BDNF/TrkB signalling has been shown to be neuroprotective in glaucomatous retinas [256,257]. Conversely, the impairment of BDFN/TrkB signalling underlies RGC and ONH vulnerability to elevated intraocular pressure-derived neurotoxicity in human and mouse retinas [258]. In addition, BDNF protects retinal explants from glutamate toxicity [259] and can stimulate upregulation of GLAST and glutamine synthetase in Müller glia, particularly under hypoxic conditions, increasing glutamate uptake [260]. In turn, intraocular injection of BDNF can further promote phosphorylation of CREB [261], in both RGCs and Müller glia, suggesting a cyclic mechanism. Interestingly, BDNF and VEGF have synergistic activity through different signalling pathways to promote neurogenesis and angiogenesis [262]. Indeed, the CAMKII/CREB pathway can be activated in Müller glia under hyperglycaemic conditions, resulting in the expression of pro-angiogenic hypoxia-inducible factor-1α (HIF-1α) and VEGF [263,264]. This, in turn, leads to the protective reestablishment of the vascular supply of neurotrophins to the retina. However, pathological angiogenesis has been associated with increased levels of HIF-1α and VEGF [265] which correlate with high levels of glutamate [266] in the vitreous humour of human patients with proliferative diabetic retinopathy. Collectively, this indicates the importance of balancing the different cellular responses to glutamate excitotoxicity. 

NMDA receptor signalling has been described as paradoxical because it can promote both neuroprotective and neurodegenerative pathways, mediated through different downstream signalling pathways. For instance, the application of NMDA to rat retinas resulted in the simultaneous activation of both pro-survival phosphatidylinositol-3 kinase (PI3K)-Akt and extracellular signal-regulated kinase (ERK) signalling as well as apoptotic activation of p38 MAP kinase and c-Jun N-terminal kinase (JNK) signalling in RGCs [267,268]. One explanation might reflect the different signalling elicited by synaptic versus extrasynaptic localised NMDA receptors [201], as mentioned earlier. It has been shown in hippocampal neurons that the activation of extrasynaptic NMDA receptors leads to degenerative cellular processes including activation of gene programmes associated with cell death. Whereas synaptic NMDA receptor signalling is associated with cell survival transcription programmes mediated by CREB and ERK phosphorylation, extrasynaptic NMDA receptor activation leads to CREB shut-off and ERK retention [205,214]. Although these alternative signalling pathways have not been demonstrated in RGCs, it was recently shown that the neurodegenerative effects of NMDA receptors are mediated by their interaction with the channel TRPM4, a protein found only in extrasynaptic locations, and that inhibition of this interaction was neuroprotective for RGCs following intraocular NMDA injection [202]. Other signalling pathways affected by prolonged NMDA receptor activation in the retina include inhibition of the PIK3/Akt/mTOR pro-survival signalling cascade following retinal ischemic–reperfusion, which was relieved upon NMDA receptor blockade [269]. The significance of this pathway is further supported by pharmacological activation of PI3K/Akt/mTOR-signalling to selectively rescue RGCs from NMDA receptor-mediated cell death in ischemic and glaucomatous retinal models [270,271]. In addition, mTOR activity has been shown to promote RGC survival and axonal regeneration [272,273] and to be necessary for the modulation of neuronal autophagy in diabetic retinopathy [274].

In summary, impaired protective signalling pathways and pathogenic changes in gene expression following prolonged NMDA receptor activation have been described in RGCs. However, a deeper insight into the consequences for organelle function is necessary to better understand subsequent cellular death mechanisms.

### 5.3. Mitochondrial Dysfunction

RGCs span from the IPL of the retina, where their dendrites make contacts with their presynaptic partners, past the GCL where their cell bodies are located, to the RNFL and the ONH. Here, the initial segments of the axons form unmyelinated bundles of fibres, which ultimately form the myelinated optic nerve which establishes postsynaptic contacts with different brain areas responsible for further processing of visual information. Hence, different compartments of a single RGC are exposed to different extracellular environments and have substantially non-homogeneous energetic demands [275]. In addition to presynaptic and dendritic localization of mitochondria [276], strong expression of mitochondrial cytochrome *c* oxidase has been shown in the GCL (where their cell bodies are located) and ONH but not in the myelinated regions of the optic nerve [277]. This reflects the high oxygen and energy demands required of RGC cell bodies and their unmyelinated axons in order to generate and transmit action potentials. However, high mitochondrial compartmentalisation in these areas also make RGCs particularly vulnerable to any imbalance in mitochondrial homeostasis [125,278,279], as has been shown in glaucoma [280,281,282], ischemic retinopathy [283,284] and diabetic retinopathy [285]. Mitochondrial homeostasis involves the balancing of respiration and ROS production, the organelle degradation rate through mitophagy-associated proteins, and the fusion and fission activity which is directly linked to the capability of mitochondrial mobilization via cytoskeleton transportation. These processes are significantly altered following NMDA receptor-induced calcium overload in RGCs, leading to the activation of pro-apoptotic cascades.

A high amount of energy is consumed by neurons in order to maintain calcium homeostasis and to quickly restore low concentrations of intracellular calcium following synaptic NMDA receptor activation [286]. Indeed, calcium is normally taken up by mitochondria through the mitochondrial calcium uniporter expressed on the mitochondrial inner membrane and locally buffered at the interface with ER membrane-located IP3Rs and RyRs [229,287]. However, prolonged NMDA receptor activation, particularly of extrasynaptic receptors, leads to saturation of the mitochondrial buffering capacity and the extrusion of calcium through the mitochondrial permeability transition pore, thus altering the mitochondrial membrane potential necessary for energy production [201,288]. In addition, the majority of RGC energy metabolism relies on oxidative phosphorylation via the electron transport chain of the mitochondria and on aerobic glycolysis similar to the Warburg effect described for certain cancers [289]. Therefore, these cells are particularly prone to oxidative stress. When the mitochondrial aerobic respiration activity is overloaded, it causes hyperpolarization of the mitochondrial membrane potential and production of ROS such as nitric oxide, which exacerbates NMDA receptor-mediated toxicity. In particular, nitric oxide has been shown to potentiate NMDA receptor-mediated toxicity in RGCs [290,291], and increased levels of nitric oxide have been shown in both diabetes [292,293] and glaucoma [294]. The coupling of NMDA receptors and neuronal nitric oxide synthase complex activity has been shown to be detrimental to neuronal survival [295], as has also been demonstrated in retinal models of glutamate excitotoxicity, hypoxia and glaucoma [296,297]. Interestingly, it was shown that the interaction between NMDA receptors and PSD-95 mediates the neurotoxic coupling of NMDA receptor over-activation with neuronal nitric oxide synthase in cortical neurons [295]. Similarly, the specific disruption of the complex between PSD-95 and GluN2B subunit rescued neurons in a mouse model of brain ischemia [298], suggesting the possible involvement of the GluN2B-containing extrasynaptic NMDA receptors [299].

NMDA receptors can also act as sensors of free radical elevation through the redox modulatory sites present on GluN1 and GluN2A subunits, and increases in ROS have been described to increase the opening probability of NMDA receptors [300]. Similarly, this site can be oxidised by redox agents such as oxidized glutathione, which also modulate NMDA receptor activity [301]. Interestingly, it has been demonstrated that the mitochondrial membrane potential can be rescued, and RGC survival increased by promoting the pro-survival signalling of synaptic NMDA receptors [302]. Collectively, these studies have shown that oxidative stress affects NMDA receptor activity and, at the same time, prolonged and extrasynaptic NMDA receptor signalling directly affects mitochondrial respiration by enhancing ROS production. Thus, similar to other neuronal types, RGCs are likely to undergo NMDA receptor-induced mitochondrial dysfunction and ROS production, which ultimately leads to the activation of pro-apoptotic pathways [299,303], as for instance, has been shown under hypoxic conditions [218]. In particular, calpain, a calcium-activated protease, has been shown to be activated in optic neuritis [304], glaucoma [305,306], and under hypoxic conditions [307] and has been described as one of the major factors that disrupt the axonal cytoskeletal architecture and ultimately commit RGCs to cell death [308,309,310,311,312]. In addition, it has been shown that glutamate-induced calpain activity promotes the translocation of the GluN2A subunit to the membrane in in vitro retinal cultures [313].

Mitochondrial turnover, which includes the biogenesis of new functional organelles, degradation of damaged organelles (a process termed ‘mitophagy’) and recycling of existing pools of organelles through fission and fusion processes, is a highly dynamic process that, if malfunctioning, can ultimately trigger apoptotic cascades. In particular, functional impairment of mitophagy-related proteins such as PINK1 (PTEN-induced kinase 1), Parkin and Optineurin has been shown to occur in both glaucoma [314,315,316] and diabetic retinopathy [317]. The relevance of these processes to glutamate excitotoxicity is demonstrated, for instance, by the overexpression of Parkin in RGCs, which protects isolated RGCs from glutamate and NMDA exposure, as well as in models of glaucoma [318,319]. Furthermore, cultured RGCs were also protected from glutamate excitotoxicity upon treatment with deubiquitinating enzyme inhibitors to modulate the Parkin-mediated mitophagy pathway [320]. Overall, DCD, consisting of a secondary and irreversible intracellular calcium increase, hence the term ‘dysregulated calcium dynamics’, has been shown to enhance mitochondrial degradation which ultimately leads to energy failure due to a decreased number of mitochondria [321]. Furthermore, neuronal survival is also dependent on the balancing of mitochondrial fission and fusion, which in turn affects microtubule-mediated mobility and actin-dependent anchoring of mitochondria at presynaptic sites and dendritic shafts [322,323]. In specific subtypes of RGCs, it has been shown that genetic mutations of fusion proteins, such as the dynamin-like protein encoded by the *OPA1* gene (OPA1 Mitochondrial Dynamin Like GTPase) gene, lead to mitochondrial fragmentation [324,325]. Interestingly, *OPA1* gene mutation in RGCs leads to increased sensitivity to glutamate-induced DCD and energy failure [326], as well as NMDA receptor upregulation, exacerbation of oxidative stress and induction of apoptotic pathways [327]. Similarly, in the DBA/2J glaucoma disease model, the downregulation of the *OPA1* gene causes mitochondrial fragmentation and the promotion of apoptotic pathways [328]. This effect can be rescued through memantine treatment, a preferential inhibitor of extrasynaptic NMDA receptors, which induces *OPA1* gene upregulation and promotes RGC survival [328]. Hence, the physiological functioning of mitochondrial turnover is coupled with NMDA receptor activity in neurons in order to dynamically meet the high energy demand of different neuronal compartments. Due to the particular vulnerability of RGCs to pathological NMDA receptor-induced calcium overload, this may impact the efficient mitochondrial turnover at various levels. 

Of note, NMDA receptor and mitochondrial function are also tightly linked to neuroinflammation because both the overactivation of NMDA receptors and mitochondrial dysfunction, including the overproduction of ROS and the impairment of mitophagy, ultimately exacerbate inflammation-related degenerative processes in the inner retina [329]. In particular, NMDA toxicity in the retina has been shown to activate the NLRP3 (nucleotide-binding domain, leucine-rich-repeat containing family, pyrin domain containing-3) inflammasome complex and potentiate neuroinflammation [213]. The NLRP3 inflammasome is primed at the interface between the mitochondria and ER membrane and enhanced upon mitochondrial dysfunction and oxidative stress conditions [330], as has been discussed for RGCs in glaucomatous retinas [329]. Therefore, RGCs can undergo a vicious circle of mitochondrial dysfunction and neuroinflammation that can be mediated by NMDA receptor activation.

### 5.4. Endoplasmic Reticular Stress

The ER is intricately involved in NMDA receptor-mediated processes of DCD and mitochondrial dysfunction, whose consequences have been described above. This results primarily from the function of the ER as an important intracellular calcium store which fundamentally contributes to NMDA receptor-induced intracellular calcium increase (via CICR) and modulates both calcium homeostasis as well as neuronal electrophysiology and bioenergetics [226,331]. Segments of the ER membrane are physically associated with regions of the mitochondrial membrane through a complex of proteins termed ‘mitochondrial associated membranes’ [332], forming local spots of high intracellular calcium buffering, including calcium reuptake operated by the Sarco-Endoplasmic Reticulum Calcium ATPase (SERCA) pump expressed on the ER membrane. Hence, ER activity is highly interconnected with mitochondrial function, with both organelles playing critical roles in neurodegenerative diseases [226,229,333]. Neuronal axons display a continuous network of ER, which couples neuronal transmission with calcium homeostasis, mitochondrial energy supply, and the balance between synthesis and degradation of lipids and proteins, and therefore functions as a key site of communication between different subcellular compartments. Thus, the ER is highly dynamic within neuronal axons, and the disruption of its tubular network organisation makes neurons more susceptible to axonal degeneration in common neurodegenerative diseases [334]. In addition, trafficking of NMDA receptors to synaptic sites is also dependent upon ER function [335] and ER growth and fission in hippocampal neurons are physiologically increased upon synaptic NMDA receptor stimulation which causes dynamic organelle reorganisation [336,337]. Nevertheless, under pathological conditions, it has been shown that prolonged activation of NMDA receptors induces the depletion of ER calcium stores which contributes to neurotoxicity and promotion of apoptotic pathways in cortical neurons and organotypic brain slices [227]. Even though little is known about physiological ER tubular network dynamic organisation and ER-mediated trafficking of NMDA receptors in RGCs, a similar mechanism of NMDA receptor-mediated ER dysfunction may well contribute to RGC degeneration in glaucoma [338] as well as ischemic [339] and diabetic [340] retinopathies.

Protein accumulation is a hallmark of common neurodegenerative diseases and occurs in RGCs in pre-symptomatic stages of Alzheimer’s disease with amyloid-β and phosphorylated tau aggregates [109], and Parkinson’s disease with α-synuclein accumulation [341,342]. Interestingly, it has been shown that Alzheimer’s disease and glaucoma share similar pathophysiological changes [343], such as the phosphorylated tau accumulation in RGC dendrites caused by elevated intraocular pressure [344]. Thus, age-related ocular diseases are characterised by retinal protein accumulation [345], against which the ER implements an ‘unfolded protein response’ (UPR), consisting of the upregulation of chaperone proteins, a reduction in the rate of protein synthesis and the enhanced degradation of misfolded proteins in order to cope with inefficient protein folding [346,347]. In particular, hypoxic damage and NMDA toxicity in RGCs cause the upregulation of the ER-resident chaperone proteins Bip and p58^IPK^ as a neuroprotective response in order to prevent the production and accumulation of misfolded proteins [348,349,350]. Nevertheless, the UPR is only effective until a level is reached where the damage caused by protein accumulation is irreversible and leads to the activation of programmed cell death [346], as has been shown in RGCs exposed to ER stress and NMDA excitotoxicity [351]. In all the above-mentioned studies, concomitant increases in the C/EBP homologous protein (CHOP) were associated with the initiation of the apoptotic caspase pathway [352], and retinal treatment with an NMDA receptor antagonist has been shown to significantly decrease CHOP activity [353] and thus, the inhibition of programmed cell death.

## 6. Final Remarks and Therapeutic Perspectives

Given the importance of glutamate neurotransmission in the retinal network, this system is acutely vulnerable to glutamate excitotoxicity, which has been implicated in many neurodegenerative diseases. In fact, this vulnerability of the retina may lend itself to being an easily detectable diagnostic readout for the prognosis of degenerative diseases. As such, disturbances in visual function have also been used to diagnose early signs of common neurodegenerative diseases, whose cognitive symptoms would otherwise be harder to detect at the early stages [354,355]. 

In addition to being a diagnostic window giving insight into systemic neurodegenerative processes, the retina also constitutes a therapeutic window. As such, it has been the first tissue where different gene therapy strategies have been implemented. Retinal diseases, such as the genetic ocular pathologies affecting PCs and the retinal pigment epithelium, have been successfully treated using this strategy [356]. Due to the inherent properties of the blood–retinal barrier, it has even been postulated that the ocular route may allow drug delivery to the brain by avoiding the complications arising from the selective permeability of the blood–brain barrier [357].

Nevertheless, treatment of retinal and common neurodegenerative diseases requires a better understanding of the molecular pathways involved. Due to the subtype variation in RGC susceptibility to degenerative processes, RGCs are an ideal group for determining the factors which underlie the likelihood that an insult will lead to neuronal degeneration or not and may reveal strategies to make highly vulnerable groups of neurons more resistant. Understanding downstream mechanisms elicited in RGCs by glutamate excitotoxicity is one such area of research that may impact our understanding of both neurodegeneration and vulnerability. Indeed, by elucidating the different cellular mechanisms activated, it has become increasingly clear that targeting a single pathway may not be sufficient and that a synergistic approach may be necessary to obtain effective neuronal protection [358].

In addition, due to the essential roles that glutamatergic transmission has in mediating network communication, new strategies have aimed to interfere with degenerative signalling whilst leaving physiological signalling unimpaired [148,214]. One strategy has been to target retinal extrasynaptic NMDA receptors (avoiding those located at the synapse) through either the application of specific inhibitors such as memantine [359,360,361], targeting the extrasynaptic NMDAR-associated subunit NR2B with substances such as nafamostat or sepimostat [362], or by interfering with degeneration-promoting interactions between the NMDA receptor and its extrasynaptic partners, as was achieved through inhibiting the NMDA receptor-TRPM4 interface [202]. Alternatively, modulation of pathways downstream of NMDA receptor activation may allow one to strengthen neuroprotective [363,364] versus apoptotic [365,366] signalling pathways in the retina. One method to achieve this is to target effector mechanisms downstream of physiological NMDA receptor activation, such as enhancing either calcium homeostasis through preservation of mitochondrial function [302,320,367] or the ER-mediated UPR response [349,368]. Finally, Müller glia may also be an important therapeutic target since their functionality is integral to protecting RGCs from glutamate receptor-induced degeneration [369,370,371]. Enhancing their ability to remove toxic glutamate or to maintain a synaptic cap to prevent glutamate spill-over may be key to blocking the activation of neurodegenerative processes before they can begin. Collectively, understanding the intricate positioning of RGCs within a complex network involving not just neurons but also Müller glia as well as the retinal vasculature not only helps to explain the vulnerability of these cells to glutamate-induced RGC degeneration, but may direct strategies to achieve neuroprotection.

## Figures and Tables

**Table 1:** Distribution of the different subunits of ionotropic and metabotropic glutamate receptors (iGluRs and mGluRs) and glutamate transporters within the rodent and primate retinal network including (in order from the outer layers to the inner layers of the retina) photoreceptors, horizontal cells, bipolar cells, amacrine cells and retinal ganglion cells. The expression profile of Müller glia, since relevant to transmission modulation, has been included as well. iGluRs are distinguished as AMPA (four subunits, GluA1-4), kainate (five subunits, GluK1-5), GluD (two subunits, GluD1-2) and *N*-methyl-d-aspartate (NMDA) (seven subunits, GluN1, 2A-D, 3A-B) receptors. mGluRs are classified as three subgroups (I-III) and comprise 8 subunits (mGluR1-8); they are all expressed (except for mGluR3) throughout the retina in different combinations and locations, and here only subunits with relevant and well-studied functions in the retina are mentioned. Glutamate transporters include the sodium-dependent excitatory amino acid transporters classified into five subtypes (EAAT1-5) and the chloride-dependent cysteine-glutamate transporter (xCT). * hinted at by pharmacological blockade, but immunocytochemical evidence is lacking.

	*Ionotropic Glutamate Receptors*			*Metabotropic* *Glutamate Receptors*	*Glutamate* *Transporters*
	AMPA Receptors (GluA1-2)	Kainate Receptors (GluK1-5)	NMDA Receptors (GluN1, 2A-D, 3A-B)	Group I-III Metabotropic Receptors (mGluR1-8)	EAAT1-5 xCT
**Photoreceptors**	-	GluK5 *only presynaptic* [28]	GluN1, 2B *only presynaptic* [29,30]	mGluR8 [31]	EAAT2 [24,32] EAAT5 [33]
**Horizontal cells**	GluA2-4 [26]	-	GluN1 [29,34]/ *additional subunits unknown*	-	EAAT3 [32,35]
**Bipolar cells**	GluA1-4 [22]	GluK1 (*OFF-BCs*) [36] GluK1-3 [22,37]	GluN1,2C-D [23,37] GluN2D *only presynaptic* in rod BCs [29,38]	mGluR6 (*ON-BCs*) [17] mGluR8 [39,40] mGluR7 *presynaptic* [40,41]	EAAT2 [24,32] EAAT3 [32,35] EAAT5 [33]
**Amacrine cells**	GluA1-4 [22,26]	GluK2-5 [22,26,37] GluD1-2 [42]	GluN1,2A-C [29,37]	mGluR1 [22] mGluR2 [43] mGluR5 [44] mGluR7 *postsynaptic* [40,41]	EAAT2 [24,32] EAAT3 [32,35]
**Retinal** **ganglion cells**	GluA1-4 [22,26]	GluK2-5 [22,26,37]	GluN1,2-C [23,26,29,37] GluN3A [45]	mGluR1 [22]	EAAT3 [32,35]
**Müller** **glia**	AMPA receptor (in vitro) [46] * GluA4 [22]	-	NMDA receptor (in vitro) [47,48] *	-	EAAT1 [24,32,48] EAAT4 [49] xCT [50]
